# Editorial: Role of lung and gut microbiota in the immune response against respiratory viral infections

**DOI:** 10.3389/fimmu.2022.1114581

**Published:** 2023-01-09

**Authors:** Ana Paula Duarte de Souza, Aran Singanayagam, Bárbara Nery Porto

**Affiliations:** ^1^ Laboratory of Clinical and experimental Immunology, School of Health and Life Science, Pontifical Catholic University of Rio Grande do Sul, Porto Alegre, Brazil; ^2^ Centre for Molecular Bacteriology and Infection, Imperial College London, London, United Kingdom; ^3^ Department of Medical Microbiology and Infectious Diseases, Rady Faculty of Health Sciences, University of Manitoba, Winnipeg, MB, Canada; ^4^ Biology of Breathing Group, Children’s Hospital Research Institute of Manitoba, Winnipeg, MB, Canada

**Keywords:** respiratory viral infection, lung microbiota, gut microbiota, virome, innate immune response, adaptative immune response

The respiratory tract is constantly exposed to potentially pathogenic microorganisms, including viruses. The most important human respiratory viruses are influenza, rhinovirus, respiratory syncytial virus, and coronaviruses. Viral clearance and the resolution of infection require a complex response triggered by immune cells. Several factors have been described to contribute to a protective immune response against respiratory viral infections, including the host microbiota.

The host microbiota and microbiota-derived metabolites act as messengers by providing local and systemic signals to control the innate and adaptive immune responses. However, the intricate interactions between the microbiota, its metabolites and the host immune system are not fully understood during respiratory viral infections. The microbiota consists of different microorganisms including bacteria, viruses, protozoa, and fungi that colonize the human body and establish important interactions with human cells. Emerging evidence indicate that the microbiota is relevant for respiratory health, playing a protective role during respiratory viral infections by triggering different immune mechanisms, including an antiviral response. This crosstalk between gut microbiota and lung immunity contributes to the maintenance of pulmonary homeostasis and is known as the gut-lung axis (1). More recently, the lung microbiota has also been identified and studies have been demonstrating its role during respiratory viral infections and the interaction with the immune response.

These connections between the lung, microbiota, and the immune system can start early in life as highlighted by Yagi et al. The first contact with the commensal bacteria initiated upon the infant’s birth helps to develop the immune system. Yagi et al. focus their review article on the role of gut and lung microbiome in protection against respiratory syncytial virus (RSV). RSV is the most common cause of hospitalization due to bronchiolitis and pneumonia in children younger than one-year of age. Yagi et al. explain how lung and gut microbiota composition can play a role in the severity of the infection and the associated immune response. For example, the dominance of *Haemophilus influenzae* and *Streptococcus* in the nasopharyngeal microbiome has been linked to interferon responses, toll-like receptor, and inflammatory genes expression and is associated with a more severe RSV-associated disease (2). Moreover, Yagi et al. underlined that therapeutic approaches associated with the microbiota can be used to prevent RSV infection by modulating the immune response. They argued that the first years of life, when the microbiota is established and when the immune system is maturing, represents an appropriate time for interventions to prevent severe respiratory infection in children. In keeping with this concept, the study of Fonseca et al. showed that maternal supplementation with the probiotic *L. johnsonii* modulates microbiota and regulates offspring immunity to RSV (3). The authors identified decreased airway mucus and reduced Th2-mediated immune responses in a model of neonatal RSV infection.

Related findings advocating the use of bacteria components for the prevention of RSV infection in mice were demonstrated in a manuscript by Antunes et al. They report that OM-85, a bacterial lysate considered a postbiotic, can prevent RSV infection when administered intranasally. The protective effect was associated with the induction of antiviral response mediated by the type 1 interferon pathway. Moreover, the authors found an increase in the alveolar macrophages, tolerogenic dendritic cells (DCs), expansion of regulatory T cells (Tregs), and Th1 preventing the ILC2 recruitment to the lungs.

Another intervention studied to prevent RSV disease in mice is the supplementation of a high-fiber diet that led to the modulation of microbiota and the immune system (4). The use of dietary fibers, known as probiotics, can induce the production of bacterial metabolites, such as short-chain fatty acids (SCFA), mainly represented by acetate, propionate, and butyrate. Many members of the microbiota obtain carbon and energy from the fermentation of complex carbohydrates, such as soluble fibers which are indigestible by the human host. The SCFA is a well-recognized link between the microbiota and host cells.

Wilson et al. reviewed how microbial metabolites, such as the SCFA, can impact the development and activation of DCs. DCs are a group of specialized myeloid cells that monitor the respiratory tract microenvironment and are responsible for protection against viruses by relaying environmental and antigenic information to T cells. Wilson et al. highlighted especially the effects of butyrate and propionate on DCs, since the majority of studies focus on these two SCFA in this context. To summarize, butyrate and propionate effects on DCs *in vivo* are related to increasing immunosuppressive phenotype, decrease in Th2 induction, and airway inflammation. Other bacterial metabolites have been described to influence DCs, such as deoxycholic acid (DCA), spermidine, p-cresol sulfate (PCS) and desaminotyrosine (DAT) (Wilson et al.).

These bacterial metabolites can play immunomodulatory roles at the local mucosal site where they are produced, but also can enter the circulation and modulate the immune response in distal tissues. The distal outcomes of mucosal infection in intestinal and lung inflammation were reviewed by Melo-Gonzalez et al. DAT, for example, has been shown to protect against influenza infection by inducing type I IFN signaling, and the administration of DAT-producing bacteria restores immunity against influenza infection in antibiotic-treated mice (5). Furthermore, respiratory viral infections, including SARS-CoV-2, have been described to impact either the intestinal microbiota or intestinal immunity through direct infection in the gut or by indirect mechanisms. Influenza virus, for example, affects gut permeability, and induce the recruitment of inflammatory lung T cells to the gut, promoting exacerbated Th17 responses and intestinal damage in a microbiota-dependent manner (Melo-Gonzalez et al.). Nevertheless, few studies have focused on how intestinal bacterial or viral infection can affect the immune response against pulmonary viral infections (Melo-Gonzalez et al.).

Since microbiota modulation can influence the immune response against by respiratory viruses, several studies have also investigated a possible role for microbiota in the immune response induced by vaccines against respiratory virus. Gonçalves et al. reviewed the findings of this growing field. Studies assessing microbiota composition and development of neutralizing antibodies have reported different results depending on the type of vaccine used. Conversely, studies analyzing antibiotic use during vaccination have shown minimal impact on the immune response induced by the vaccines against influenza. Gonçalvez et al. reviewed studies using prebiotics and probiotics as interventions to improve vaccine immune response, and the response can be variable since changes in the probiotic composition alters the specificity of the improved response. They concluded that future studies are needed to validate microbiota interventions to improve immunity during vaccination as a means of protecting against respiratory infections.

More recently, the study of the human lung virome (commensal viruses resident at mucosal surfaces) has been emerging and its possible function in the immune response against respiratory viral infections was reviewed in detail by Porto. The lung virome has been shown to have a beneficial role by stimulating a continuous local antiviral response. However, Porto highlights that the virome can also be associated with a harmful outcome, since higher viral loads can lead to detrimental consequences to the host and exacerbate disease pathogenesis, either infectious or non-infectious.

In conclusion, the microbiota can play an important role in mediating the immune response during respiratory virus infections. Several mechanisms have been proposed to explain the complex interactions between the microbiota and the immune system. Therapeutic modulation of microbiota can be an opportunity to intervene to boost the immune response during respiratory virus infection. This could have wide implications for both adults and in the early years of life.

## Author contributions

AD wrote the manuscript, AS and BP reviewed the manuscript. All authors contributed to the article and approved the submitted version.
